# Temperature-controlled power modulation compensates for heterogeneous nanoparticle distributions: a computational optimization analysis for magnetic hyperthermia

**DOI:** 10.1080/02656736.2018.1538538

**Published:** 2018-12-12

**Authors:** Sri Kamal Kandala, Eleni Liapi, Louis L. Whitcomb, Anilchandra Attaluri, Robert Ivkov

**Affiliations:** a Department of Mechanical Engineering, Whiting School of Engineering, Johns Hopkins University, Baltimore, MD, USA;; b Department of Radiation Oncology and Molecular Radiation Sciences, School of Medicine, Johns Hopkins University, Baltimore, MD, USA;; c Department of Radiology and Radiological Sciences, Johns Hopkins Hospital, Baltimore, MD, USA;; d Institute for NanoBioTechnology, Johns Hopkins University, Baltimore, MD, USA;; e Department of Oncology, School of Medicine, Johns Hopkins University Baltimore, MD, USA;; f Department of Mechanical Engineering, The Pennsylvania State University - Harrisburg, Middletown, PA, USA;; g Department of Materials Science and Engineering, Whiting School of Engineering, Johns Hopkins University, Baltimore, MD, USA

**Keywords:** Hyperthermia, magnetic nanoparticles, thermal dose, Pennes bioheat equation, perfusion modeling, bioheat transfer

## Abstract

**Purpose:**

To study, with computational models, the utility of power modulation to reduce tissue temperature heterogeneity for variable nanoparticle distributions in magnetic nanoparticle hyperthermia.

**Methods:**

Tumour and surrounding tissue were modeled by elliptical two- and three-dimensional computational phantoms having six different nanoparticle distributions. Nanoparticles were modeled as point heat sources having amplitude-dependent loss power. The total number of nanoparticles was fixed, and their spatial distribution and heat output were varied. Heat transfer was computed by solving the Pennes’ bioheat equation using finite element methods (FEM) with temperature-dependent blood perfusion. Local temperature was regulated using a proportional-integral-derivative (PID) controller. Tissue temperature, thermal dose and tissue damage were calculated. The required minimum thermal dose delivered to the tumor was kept constant, and heating power was adjusted for comparison of both the heating methods.

**Results:**

Modulated power heating produced lower and more homogeneous temperature distributions than did constant power heating for all studied nanoparticle distributions. For a concentrated nanoparticle distribution, located off-center within the tumor, the maximum temperatures inside the tumor were 16% lower for modulated power heating when compared to constant power heating. This resulted in less damage to surrounding normal tissue. Modulated power heating reached target thermal doses up to nine-fold more rapidly when compared to constant power heating.

**Conclusions:**

Controlling the temperature at the tumor-healthy tissue boundary by modulating the heating power of magnetic nanoparticles demonstrably compensates for a variable nanoparticle distribution to deliver effective treatment.

## Introduction

Hyperthermia involves raising and sustaining the temperature of malignant tumors and adjacent tissues to about 41–46 °C for ~0.5 to 2 h to achieve a therapeutic effect [1,2]. The principal challenges encountered with all thermal therapies are to effectively deliver and control energy deposition. Magnetic nanoparticle hyperthermia (mNPH) has emerged as a treatment modality that offers benefits because the heat sources, i.e., nanoparticles, can be embedded within the target tissue [3,4]. The region containing the magnetic nanoparticles is then exposed to an alternating magnetic field (AMF) which generates heat via magnetic hysteresis loss [3,5–9]. Heat generated by the nanoparticles is transferred by convective and conductive processes to the tumor, while offering the potential to minimize energy deposition outside the treatment margins. Thus, mNPH can overcome some limitations for hyperthermia that exist with other heating technologies because the energy is generated and controlled within the tumor.

While offering advantages, mNPH also presents challenges to clinical implementation. Nanoparticle delivery can be variable, and the resulting nanoparticle distribution within tumors is often heterogeneous [10–14]. The heterogeneity of nanoparticle distributions thus presents new challenges for precise energy delivery which can lead to off-target heating of healthy tissues or under-heating of regions within the tumor [14]. It has been recognized that clinical acceptance of mNPH has been slowed, in part, by the imprecise spatial control of the nanoparticles and energy within the tumor [10–13].

Computational models offer benefits to efficiently explore strategies for mNPH that consider nanoparticle distributions to identify opportunities for treatment [14–29]. Considerable progress has been made to model various complex biological and physical factors relevant to mNPH. Most models (1) assume homogeneous or regular nanoparticle distributions within the model tissue to reduce computational complexity; and, (2) neglect complexities of variable capillary blood perfusion, an important physiologic response to temperature that dissipates heat energy [15,17,24–29]. Typically, the focus of computational studies of mNPH is to determine optimal magnetic field conditions or magnetic nanoparticle parameters, i.e., specific loss power (SLP), to achieve a target temperature. Capillary blood perfusion, when included in computational studies for thermal therapy applications is typically assumed a non-physiologically constant value.

Effective hyperthermia is achieved when heat energy is controlled to minimize damaging energy deposition outside the target tissue while also achieving and maintaining an elevated temperature within the tumor. Controlling the temperature at the tumor-healthy tissue boundary offers potential to preserve normal tissue while simultaneously enabling therapeutic heating within tumors. However, controlling heat deposition with temperature-feedback control at the tumor-tissue boundary may limit therapeutic heating throughout the tumor when the nanoparticle distribution is inhomogeneous [14]. Furthermore, locally high nanoparticle concentrations deposit sufficient energy to generate ‘runaway’ heating with local ablation and excess energy deposition that threatens to overheat adjacent normal tissues. Thermal dose regulation requires a responsive control system to rapidly modulate energy deposition with appropriate temperature feedback.

Proportional-integral-derivative (PID) control systems are widely used in industry and in medicine to regulate input power for heating [30–34]. Salomir et al. used a PID temperature controller to regulate the temperature at a focal point during MR-guided focused ultrasound therapy [30]. They obtained temperature control at nearly the precision of the temperature measurement, *in vitro* and *in vivo*. Mougenot et al. further developed three-dimensional spatial and temporal control of temperature during MR thermometry-guided focused ultrasound therapy [31]. Haemmerich et al. implemented an automatic PI closed-loop controller system and optimized the controller parameters for temperature controlled RF ablation [32].

Here, we describe a strategy to modulate AMF amplitude to regulate nanoparticle SLP using single-point temperature feedback with a PID controller to compensate for variable nanoparticle distributions. Our computational 2D and 3D phantoms incorporated a temperature-dependent capillary blood perfusion model that reduces with significant thermal dose. We considered six nanoparticle distributions and we compared time-dependent changes of temperature and thermal dose, the latter defined by cumulative equivalent minutes at 43 °C (CEM43). In our simulated models, we study the use of PID-controlled power modulation to reduce temperature heterogeneity in the tumor; and, to achieve a more rapid approach to the therapeutic thermal dose compared to constant power heating.

## Materials and methods

### Nanoparticle distributions

**Figure 1.**
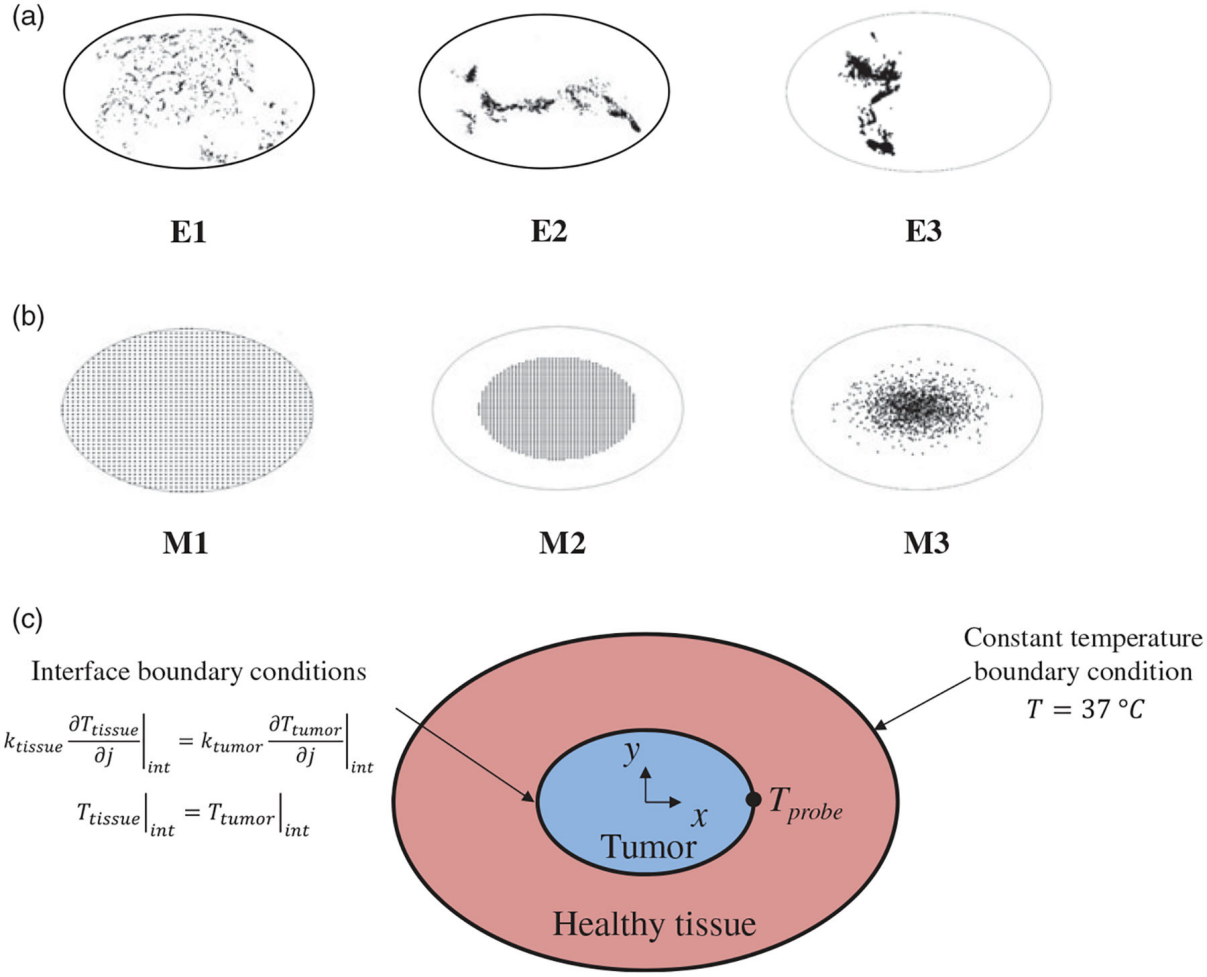
Simulated nanoparticle distributions and schematic of the computational tumor model. (a) Digitized images (processed with MATLAB) of nanoparticle distributions obtained from stained tissue sections (images previously published and used with permission, see Ref. [14]) that were extracted from human tumor xenografts obtained from nude mouse tumor models. E1-nanoparticles relatively uniformly distributed; E2-concentrated distribution along the major axis; E3-concentrated and offset along the minor axis. (b) Idealized nanoparticle distributions generated mathematically (see text for details). M1 – uniform; M2 – uniformly concentrated in 40% of tumor area; and M3 - Gaussian. (c) Schematic of the model geometry, tumor region surrounded by the healthy tissue, with thermal boundary conditions. Note: Figures not to scale.

In the present study, the magnetic nanoparticles were modeled as point heat sources [20]. Three distributions – E1, E2, and E3 in Figure 1(a) - were generated by digitizing (processed with MATLAB) images previously published from tissue sections obtained from human prostate tumor xenografts grown in mice which were injected with BNF-Starch (micro-mod Partikeltechnologie, GmbH, Rostock, Germany) nanoparticles [35]. An additional three were mathematical functions representing ‘idealized’ distributions – M1, M2, and M3 shown in Figure 1(b), and described below. The heating power of the phantom nanoparticles was modeled using previously reported specific loss power (SLP) values for magnetic iron oxide nanoparticles (see Figure S1) [36]. The corresponding alternating magnetic field amplitude can be estimated using the continuous polynomial approximation of SLP vs *H* given by Soetaert et al. [29].

To compare heating effects among the six tumor models, the total number of nanoparticles N (dark pixels) in a tumor was fixed at *N* = 1460 ±5 for each model [29]. For the present computational analysis, the area of a phantom ‘nanoparticle’ was fixed at ~35 pixels/mm^2^ in the 2D models, as analyzed from imaging data obtained from a previous study and corresponding approximately to the known injected concentration of nanoparticles into human tumor xenografts (= 5 mg Fe/cm^3^ of tumor) as reported in that study [14]. This means that each phantom tumor received a total heating power comparable to the others, allowing a quantitative comparison of temperatures and thermal dose. If the individual heating power of a phantom nanoparticle is *Q*_*i*_ (=384.8 W-nanoparticle^−1^·m^−3^), and the total number of nanoparticles *N* (= 1460) distributed in the tumor is known (see Supplementary Materials for details of discretization of heating power and Figure S1), the total heating power *Q*_NP_ power generated by the nanoparticles deposited in the tumor is then:

(1) $$Q_{NP}=\sum_{i=1}^{N}Q_{i}=5.18\times 10^{5}\text{W}/{\text{m}}^{3}$$

### Idealized nanoparticle distributions

The three mathematical nanoparticle distributions were uniform (M1) [16], centrally-concentrated (M2) [14], and Gaussian (M3) [15,28,29] (Figure 1(b)). The M1 distribution contained nanoparticles uniformly distributed throughout the tumor area. For the M2 distribution, nanoparticles were arranged uniformly in an area occupying only the central 40% region of the tumor. For M3, the nanoparticle distribution was governed by a Gaussian probability function originating at the tumor center and extending to the tumor-tissue boundary. The Gaussian probability function in Cartesian coordinates is

(2) $$P(x,y)=\frac{1}{2\pi \sigma_{x}\sigma_{y}}e^{\left(-\frac{1}{2}\left(\frac{x^{2}}{\sigma_{x}^{2}}+\frac{y^{2}}{\sigma_{y}^{2}}\right)\right)}$$

where *σ*_*x*_ = 2 (~44% of tumor major semi axis) and *σ*_*y*_ = 0.5 (~17% of tumor minor semi axis) are arbitrarily selected standard deviations of *x* and *y*, respectively. The generated Gaussian distribution of points was truncated to fit within the tumor.

### 2D tumor model

We chose to develop, test and compare our strategy with constant power heating using a 2D tumor model. This approach helps us to efficiently test our strategy before translating our results to 3D and is recommended by American Society of Mechanical Engineers (ASME) [37–39]. Our chosen 2D model comprised two concentric elliptical regions, shown in Figure 1(c), representing the tumor and surrounding healthy tissue. The inner elliptical region (major axis *l*_*t*_ = 9 mm, minor axis *w*_*t*_ = 6 mm) represented the tumor seeded with nanoparticles. The outer elliptical region (major axis *l* = 19 mm, minor axis *w* = 16 mm) corresponded to modeled healthy tissue. We assumed that the outer boundary of the healthy tissue was in contact with the core body temperature (infinite heat reservoir) maintained at 37 °C. Heat transfer in the healthy tissue and tumor can be described by the Pennes’ bioheat equation as [40]

(3) $$\rho_{n}c_{n}\frac{\partial T_{n}(x,y,t)}{\partial t}=k_{n}\nabla^{2}T_{n}(x,y,t)+\rho_{b}c_{b}\omega_{b,n}\left(x,y,T_{n}\right)\left(T_{b}-T_{n}\left(x,y,t\right)\right)+Q_{m,n}+Q_{P}$$

where the subscript *n* accounts for the tissue layer (*n* = 1 for the tumor and *n* = 2 for healthy tissue) and the subscript *b* for blood, respectively. ρ_*n*_ is the respective tissue density (kg/m^3^), *c*_*n*_ the respective tissue specific heat (J/kg K), *T*_*n*_(*x*, *y*, *t*) the local tissue temperature (K), *k*_*n*_ its thermal conductivity (W/m-K), *Qm*,*n* the metabolic heat generation rate in either the healthy tissue or tumor (W/m^3^), and *Q*_*P*_ is the heat rate per unit volume of tumor (W/m^3^) that arises from nanoparticles if present in that volume (= 0 if no phantom nanoparticle is present). The subscript m in Equation (3) refers to metabolic heat generation in tissue layer *n*. ρ_*b*_, *c*_*b*_, ω_*b*_, _*n*_(*x*, *y*, *T*_*n*_) and *T*_*b*_ denote density, specific heat, perfusion rate, and temperature of blood, respectively. Table 1 summarizes the thermophysical properties used in this study [41–46]. We assumed no power deposition due to eddy currents by AMF interactions with tissue.

At the interface between the tumor and healthy tissue, continuity in temperature and heat flux is given by (see Figure 1(c)):

(4) $$k_{tissue}\frac{\partial T_{tissue}}{\partial j}{|}_{int}=k_{tumour}\frac{\partial T_{tumor}}{\partial j}{|}_{int}$$

(5) $$T_{tissue\;int}=T_{tumour\;int}$$

In Equation (4), *j* denotes the surface normal to the elliptical boundary between tumor and healthy tissue. Initial temperature of tumor and healthy tissue was set to the core body temperature, i.e., *T*_*tissue boundary*_ = 37°C.

### Perfusion models

Effects of temperature on perfusion were tested using three models: (i) constant perfusion (i.e., ‘classic’ Pennes’ equation) as described by Equation (3), where ω_b_(*T*) = ω_*bi*_ (1/s); (ii) Arrhenius perfusion; and, (iii) modified Arrhenius perfusion, as described previously [47,48]. In the Arrhenius model, blood perfusion is described as a function of temperature and time, correlated to the degree of microvascular stasis (DS). DS is a dimensionless value expressed as

(6) $$\text{DS}=1-\text{exp}\left(-A\int_{0}^{t}e^{-E_{a}/RT(\tau )}\right)d\tau$$

with

(7) $$\omega_{b}(T)=\omega_{bi}\left(1-DS\right).$$

In Equations (6) and (7), *A* is the frequency or pre-exponential factor (1/s), *E*_*a*_ the activation energy (J/mol), *R* the universal gas constant (J/K·mol), *T*(*τ*) is the absolute tissue temperature as a function of time and ω_*bi*_ the initial blood perfusion rate (1/sec). The degree of vascular stasis *DS* varies between 0 (no vascular damage) and 1 (complete vascular shutdown). Values for *A* and *E*_*a*_ are shown in Table 1.

The foundation of the modified Arrhenius perfusion model is the first-order kinetic Arrhenius model of vascular stasis (Equations (6) and (7)), expanded by an additional term to increase the relative perfusion when vascular stasis is low. This latter model is derived empirically from data determined experimentally by He et al. [47]. Schutt et al. represented the resulting curve by four linear segments to improve computational efficiency, yielding [48]

(8) $$\omega_{\text{b}}(\text{T})=\{\begin{array}{c} \omega_{\text{bi}}(30.\text{DS}+1),\;(DS\leq 0.02)\\ \omega_{\text{bi}}(-13\text{ DS}+1.86),\;(0.02<DS\leq 0.08)\\ \omega_{\text{bi}}(-0.79\text{ DS}+0.884),\;(0.08<\text{DS}\leq 0.97)\\ \omega_{\text{bi}}(-3.87\text{ DS}+3.87),\;(0.97<\text{DS}\leq 1.0). \end{array}$$

When the degree of stasis is low (*DS* ≤ 0.02), there is an increase in perfusion. As the degree of stasis increases (0.02 < *DS* ≤ 0.08), perfusion first decreases moderately, and then more rapidly (0.08<*DS* ≤ 0.97), before eventually becoming zero (0.97<*DS* ≤ 1.0).

### Constant power heating

For constant power simulations, the total heating power of nanoparticles, *Q*_NP_, was fixed. Temperature distribution *T*(*x*, *y*, *t*) and thermal dose were evaluated as a function of time for each simulation. The total thermal dose achieved in the tumor is expressed as [50]:

(9) $$CEM43=\int_{t=0}^{t=final}B^{\frac{43^{\circ }C-T\left(x,y,t\right)}{1^{\circ }C}}dt$$

In Equation (9), *B* is a constant with value 0.5 for *T*> 43 °C, and 0.25 for *T*≤ 43 °C [1]. CEM43 T90 is defined as the total isoeffect thermal dose expressed in cumulative equivalent minutes at 43 °C achieved or exceeded in 90% of the tumor area [1,51], with treatment considered effective when achieved for 60 min or longer, i.e., CEM43 T90 ≥ 60 [1,51]. The power required to achieve CEM43 T90 ≥ 60 within 20 min was computed. The heating power needed to achieve the target dose was defined as the isoeffect heating power, *Q*_iso_. It was computed by iteratively performing simulations with constant power settings until CEM43 T90 ≥ 60 was achieved.

### Modulated power heating

**Figure 2.**
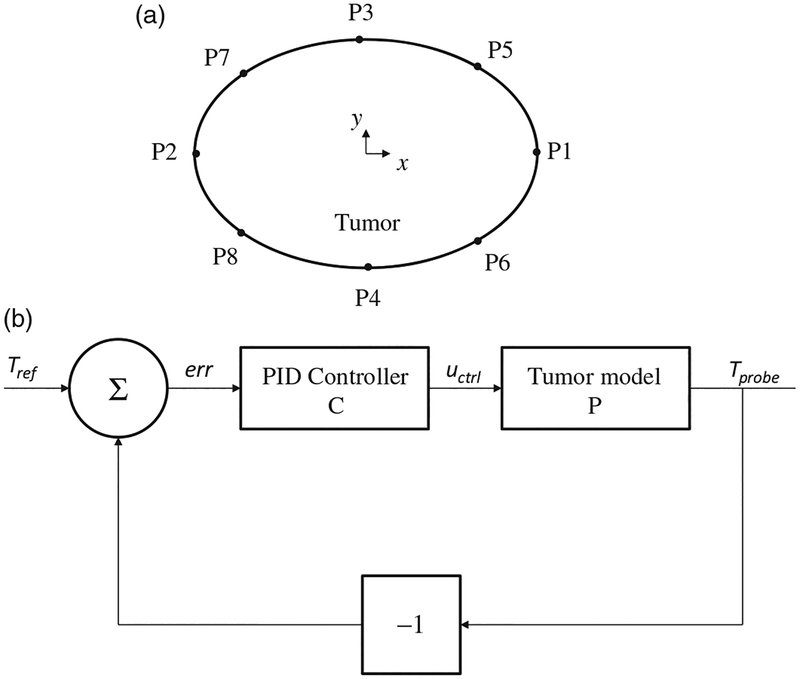
Modulating power based on single-point temperature feedback, choice of probe location and power modulation algorithm. (a) Eight locations on the tumor-tissue boundary were tested for temperature feedback during power modulated heating using criteria described in text. (b) Block diagram of feedback loop with the PID (proportional-integral-derivative) controller for modulating nanoparticle heat output.

For modulated-power heating, nanoparticle heating was varied as a function of the temperature computed at the tumor-tissue boundary [19,24]. Attaluri et al. have previously described methods to heat tumors using temperature-control at the tumor-tissue boundary to control energy deposition for a spherical tumor model with uniform distribution of nanoparticles [14]. Presently, we consider a 2D elliptical model, which accounts for variability in two directions, and different nanoparticle distributions. These modifications demand a more precise location of the temperature probe. To determine this, eight probe locations (P1–P8, Figure 2(a)) on the tumor-tissue boundary were considered. For all six models, the heating power was varied between two levels: a higher heating power when the probe temperature, *T*_probe_ <43.5 °C, and a lower heating power when *T*_probe_ >43.5 °C [14]. The heating algorithm is described mathematically as,

(10) $$Q_{NP}\left(T\right)=\{\begin{array}{cc} 1.5\times Q_{iso}, & if\;T_{probe}<43.5^{\circ }C\\ 0.15\times Q_{iso}, & if\;T_{probe}\geq 43.5^{\circ }C \end{array}$$

The higher heating power was chosen to be greater than *Q*_iso_ to achieve a rapid rise to target temperature in the tumor, which is clinically desired. A probe location that achieved a thermal dose of CEM43 ≥ 60 in 90 ±5% of tumor area and simultaneously limited the thermal dose in surrounding normal tissue to ≤5% was chosen for further temperature feedback control optimization.

For optimization with a controller, we aimed to achieve a target temperature of 43.5 °C at the probe location rapidly, and maintain it at that temperature for the remainder of treatment. The block diagram for the feedback loop of the PID (proportional-integral-derivative) temperature controller is shown in Figure 2. The temperature computed at the probe location *T*_probe_ was input into the controller with the reference temperature *T*_ref_. The difference between *T*_probe_ and *T*_ref_ input into the controller and the controller output were then substituted into the model. The closed-loop transfer function (*Tr*) for the closed-loop system (Figure 2) is given by

(14) $$Tr\;=\frac{PC}{1+PC}$$

where *P* is the plant transfer function and *C* is the controller function. For the controller, we used the general PID temperature control [34].

A proportional controller enables increasing the heating power proportionally to the difference between the computed temperature at the probe location and the target temperature [52]. In general, an integral controller ensures that the control output agrees with the target set-point temperature in steady state [34,52,53]. While 90% of controllers used in industry employ a PI controller, adding derivative control ensures closed-loop stability and enables more rapid rise to target temperature at the target location while minimizing temperature overshoot [34,52,53]. The PID controller is described by [52]

(15) $$u_{ctrl}(T)=K_{p}\theta (x,y,t)+K_{i}\int_{0}^{t}\theta (x,y,t)dt+K_{d}\frac{\partial \theta (x,y,t)}{\partial t}$$

(16) $$Q_{NP}\left(T\right)=u_{ctrl}\left(T\right)Q_{max}$$

In Equation (15), *θ*(*x*,*y*,*t*) is the difference between the computed temperature at the probe location, *T*_probe_ (*x*,*y*,*t*), and the target temperature, *T*_ref_. The gains *K*_p_ (1/K), *K*_i_ (1/(s·K)), *K*_d_ (s/K) are the proportional, integral and derivative gains of the PID controller, respectively. In Equation (16), *u*_ctrl_ adjusts the heat generation of nanoparticles, *Q*_*np*_(*T*), by modulating the heating power applied based on the difference in measured temperature at the probe location and target temperature. *Q*_max_ is the maximum power that can be generated by the nanoparticles in the model, which depends on the SLP of the nanoparticles and number of nanoparticles. The maximum SLP of 537 W/g Fe (peak field strength of 47kA/m at frequency 150 kHz) for BNF nanoparticles reported by Bordelon, et al. was used to calculate *Q*_max_ (see Figure S1) [36].

A first order low pass filter was added to the derivative control to limit high-frequency noise amplification. The transfer function for the controller *C*(*s*) is given by,

(17) $$C(s)=K_{p}+K_{i}\frac{1}{s}+K_{d}\frac{s}{\tau_{d}s+1}$$

where *τ*_d_ is the filter time constant [33,52] given by

(18) $$\tau_{d}=\frac{1}{2\zeta f}$$

where *f* is the closed-loop frequency and ζ is the damping ratio. The cut-off frequency (1/*τ*_d_) for the low pass filter was chosen to be 4 rad/s [33,52].

The PID controller gains *K*_p_, *K*_*i*_, *K*_d_ were determined by using model control design methods based on Youla parametrization (*Q*-parametrization) [33,34,52,54]. By *Q*-parametrization theory, for a single-input single-output stable plant transfer function *P(s)*, the family of all stabilizing controllers *C(s)* can be expressed as,

(19) $$C(s)=\frac{Q(s)}{1-P(s)Q(S)}$$

where *Q(s)* is any stable transfer function. We approximated our model system by a second order plant transfer function *P*(s) with three parameters, given in Laplace domain by,

(20) $$P(s)=\frac{g}{\left(1+\tau_{1}s\right)\left(1+\tau_{2}s\right)}$$

where *g* is the static gain for step input, and *τ*_1_ and *τ*_2_ are time constants. The plant transfer function parameters were assessed by an open loop step response. The power was stepped to 30% of maximum power and the temperature response was recorded at the probe location. The static gain, *g*, is given by the ratio of temperature gain achieved with the step in control input

(21) $$g=\frac{\Delta T}{u_{ctrl}}$$

The time constant, *τ*_1_, was calculated as the delay between the maximum rate of change in temperature and the temperature response at the probe location. The time constant, *τ*_2_, is the difference between the time taken for the temperature response to reach 63% of total temperature gain and the time constant *τ*_1_ [33,52]. The relationship among these parameters and criteria for their selection has been well described by Ebert et al. [33] and visually characterized in Reference [52].

A second-order transfer function, *Tr*, was chosen, given in the Laplace domain,

(22) $$Tr\left(f,\zeta \right)=\frac{f^{2}}{s^{2}+2\zeta fs+f^{2}}$$

Using Equations (14), (19) and (22), the controller function *C*(s) was determined as,

(23) $$C(s)=\frac{f^{2}\left(\tau_{1}s+1\right)\left(\tau_{2}s+1\right)}{gs(s+2\zeta f)}.$$

The individual gains can then be calculated as,

(24) $$K_{p}=\frac{f}{2\zeta g}\left(\tau_{1}+\tau_{2}-\frac{1}{2\zeta f}\right)$$

(25) $$K_{i}=\frac{f}{2\zeta g}$$

(26) $$K_{d}=\frac{f}{2\zeta g}\left(\tau_{1}-\frac{1}{2\zeta f}\right)\left(\tau_{2}-\frac{1}{2\zeta f}\right).$$

After determining the PID controller gains, the power was modulated using temperature feedback from the probe. The computed temperature at the probe location, *T*_probe_, was compared with a reference temperature signal *T*_ref_ and the difference was input into the PID controller. In the study, the reference temperature signal *T*_ref_ was chosen to be ramp signal, beginning at 37°C and *t* = 0s, and achieving the target temperature at *t* = 30 s. This time point was chosen because it is clinically desirable to reach the target temperature rapidly. The output from the PID controller *u*_ctrl_ modulated the nanoparticle heat output.

### 3D models

**Figure 3.**
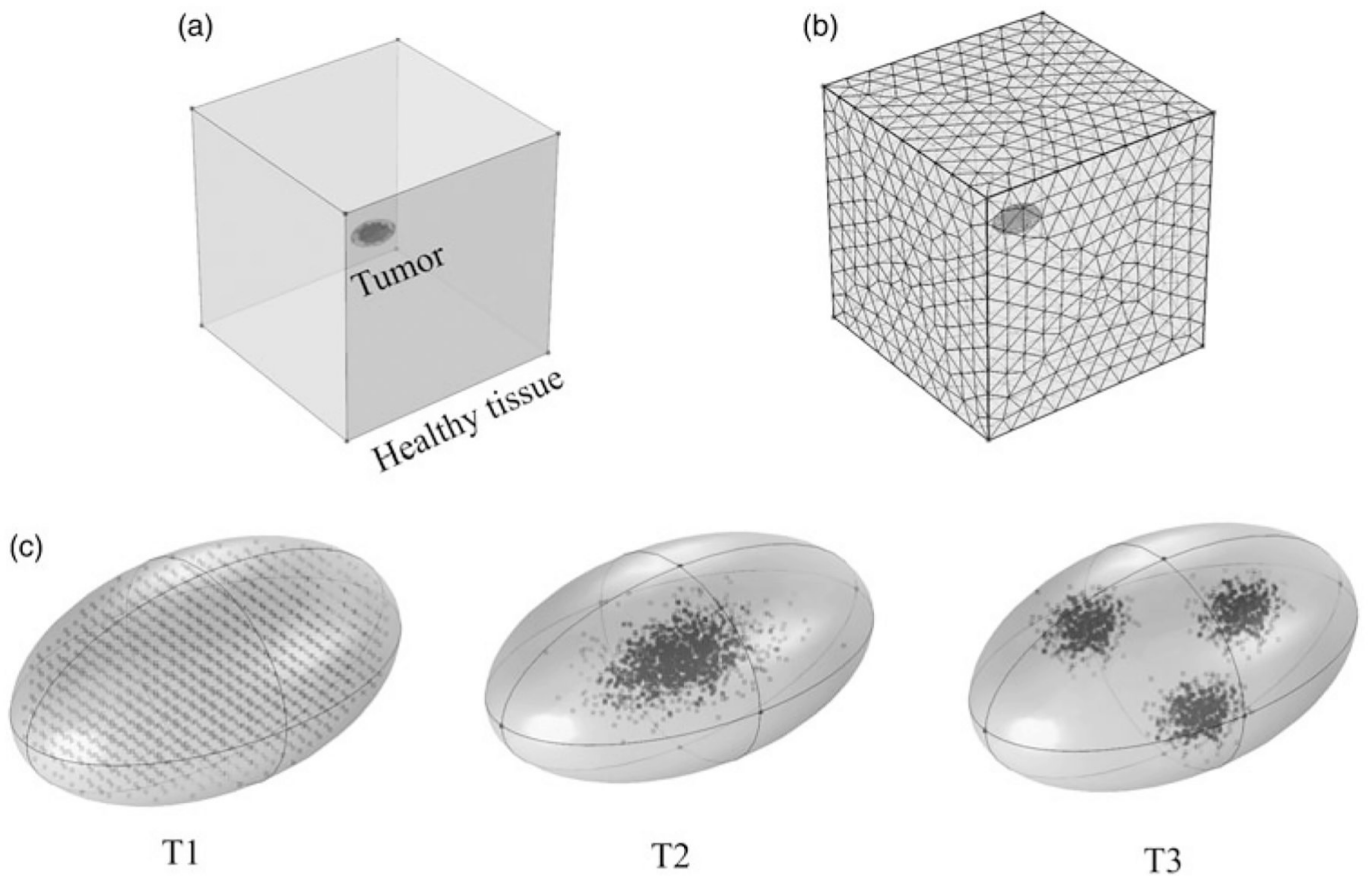
Implementation of power modulated heating using PID controller in a 3D model. (a) Schematic of the 3D computational model with tumor and surrounding healthy tissue. (b) Sample mesh for the 3D model. (c) Three nanoparticle distributions are illustrated: T1-uniform, T2 – Gaussian centered, and T3 – 3 pt-Gaussian distribution mimicking 3-point nanoparticle injection.

The healthy tissue was represented by a cube of length 5 cm (see Figures 3(a,b)). Tumors were represented by 3D ellipsoids of a total volume of 150 mm^3^ (see Figure 3(c)). Three 3D nanoparticle distribution models were considered. The T1 (uniform) distribution model contained homogeneously distributed nanoparticles throughout the tumor. In the T2 (Gaussian-centered) model, nanoparticles were normally distributed about the tumor center. For the T3 (3 pt-Gaussian), nanoparticles were normally distributed about three centers (indicating points of injection) [14]. The same thermal model that was implemented for the 2D models was used.

### Solution method

COMSOL Multiphysics 5.2a (COMSOL Multiphysics, Natick, MA), a commercial finite element solver, was used to solve numerically the governing differential equations (Equation (3)) subject to the boundary conditions described by Equations (4) and (5). Sensitivity analysis for mesh size and time-step discretization was carried out for all the 2D and 3D models to ensure solution accuracy. For example, the mesh used to compute heating for the E2 model consisted of 13 932 triangular elements with a maximum element size of 2.47 mm and a minimum element size of 0.11 mm. To check the sensitivity of the solution to mesh size, the mesh size was increased to 19 060 elements, which resulted in a change in maximum temperature of less than 0.01%. Similarly, for the transient solution, changing the time step from 1s to 0.5 s had negligible (<0.01% change) effect on the maximum temperature. Similarly, for the 3D model T2 (Gaussian-centered) model, increasing the mesh size from 17,193 tetrahedral domain elements to 23,793 tetrahedral domain elements resulted in less than 0.03% change in the maximum temperature. For the transient solution, changing the time step from 1 s to 0.1 s resulted in no significant difference (<0.001%) in the solution. Thus, the chosen grid size and time step were deemed adequate for all the 2D and 3D models. A parallel direct sparse solver (PARDISO) [55] was used for solving the partial differential equations and a gen-eralized-*α* solver [56] was used for the transient solution.

## Results

### Temperature distributions in 2D models

**Figure 4.**
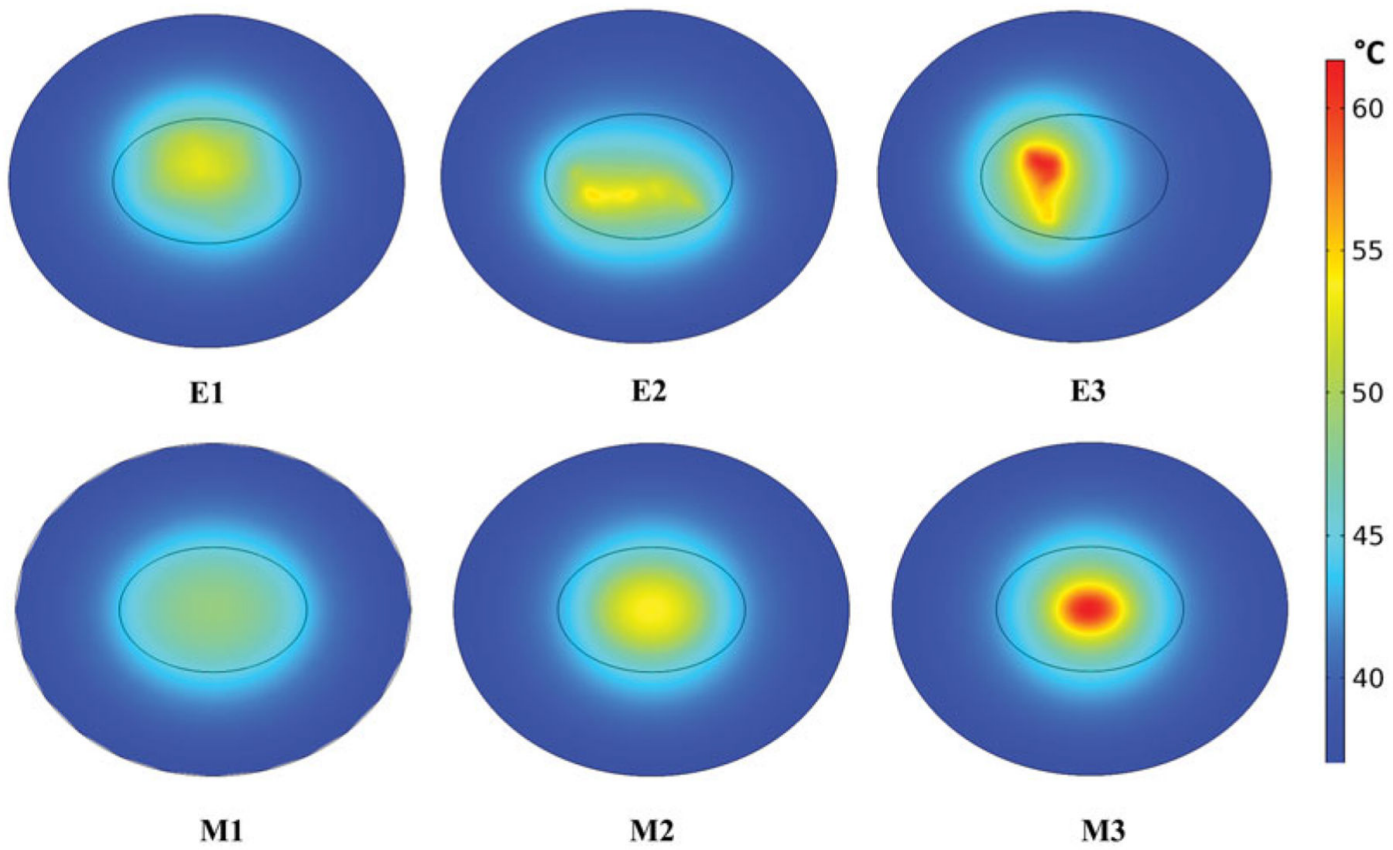
Localized nanoparticle distributions lead to higher temperatures with constant perfusion model and constant power heating. Computed temperature distributions in the tumor and surrounding healthy tissue for six nanoparticle distributions after 20 min of heating at constant power *Q*_NP_ = 10.6×10^5^ W/m^3^ with constant perfusion.

Temperatures achieved in the 2D models of tumor and healthy tissues after 20 min of constant-power heating and constant perfusion are shown in Figure 4, and plotted graphically in Figure S2(a). Values used in the calculations are provided in Table 1. Constant power and constant perfusion results provided a reference against which to compare various strategies. A constant power of *Q*_NP_ = 10.6 × 10^5^ W/m^3^ was chosen because it yielded a minimum thermal dose of CEM43 ≥ 60 min in 90% area of tumor for the uniform (M1) model, which served as our chosen reference. The M1 model exhibited the lowest range of temperature variations (~43–49 °C), and lowest overall temperatures, see Figures 4 and S2 (a). By contrast, the idealized Gaussian nanoparticle (M3) distribution produced the highest maximum temperature, and the E3 model exhibited the highest variation of temperatures. The power required to achieve equivalent thermal dose, i.e., isoeffect power with M1 as the reference was greater in all other models, see Table 2.

When temperature-dependent perfusion was considered, the range of tumor temperatures increased for all nanoparticle distributions compared to constant perfusion, and there was little difference between the two temperature-dependent perfusion models (see Figures S2 (b) and (c)). Conversely, by including temperature-dependent perfusion less power was required to achieve the target thermal (isoeffect) dose, and thermal dose to normal tissues was decreased for the off-center, concentrated nanoparticle distribution (E3), but changed little for other nanoparticle distributions (see Table 2).

### Modulated power heating

Single-point thermometry at the simulated tumor-tissue boundary was used to provide data to modulate power during heating. The probe location for further optimization simulation with PID control was selected by simulating heating in each model using a two-step power function (see Equation (10) at each location. The probe location that predicted a thermal dose conforming to our chosen criteria was selected (see Table 3). Probe positions for distribution models M1 and M2 were identical due to symmetry. However, for the off-center concentrated nanoparticle distribution, E3, no location satisfied both criteria simultaneously. In this case, the location P6 (see Figure 2 and Table 3) was chosen.

PID control was implemented by computing the open loop gains for each of the nanoparticle distribution models (Table 4). Of the six nanoparticle distributions, the uniform distribution model (M1) had the highest open loop gain *g* (23.8 K) while the E3 model had the least gain (17.6 K). Nanoparticles located closer to the probe produced higher open loop gains, while models having the probe located farther away from the nanoparticles generated lower open loop gains. Distributions with higher open loop gains required lower proportional gains, with M1(uniform) model having the lowest proportional gain (7.4 1/K) and E3 model having the highest proportional gain (14.9 1/K). Since the derivative controller allows for higher proportional gains, models with higher proportional gain have higher derivative gains (for E3, *K*_d_ = 145.54 s/K).

The time-dependent power application differed between dispersed and concentrated nanoparticle distributions (see Figure S4). For dispersed nanoparticle configurations, M1 and E1, initial power was at maximum and then decreased to a lower value for the remainder of heating. For distributions having concentrated regions of nanoparticles (i.e., E2, E3 and M2, M3), the applied power initially increased to maximum for a period of time, and then oscillated between a selected maximum and minimum for the duration of heating.

The results of simulated tissue heating are displayed graphically in Figure S5, which also contains tissue damage (a) calculations. High nanoparticle concentrations, located off-center within the tumor and proximal to a normal tissue boundary represent the least ideal treatment scenario (Figure S4). It is worth noting that in all cases the ideal probe location was farthest from the nanoparticle clusters.

### Comparing constant power with PID controlled heating

**Figure 5.**
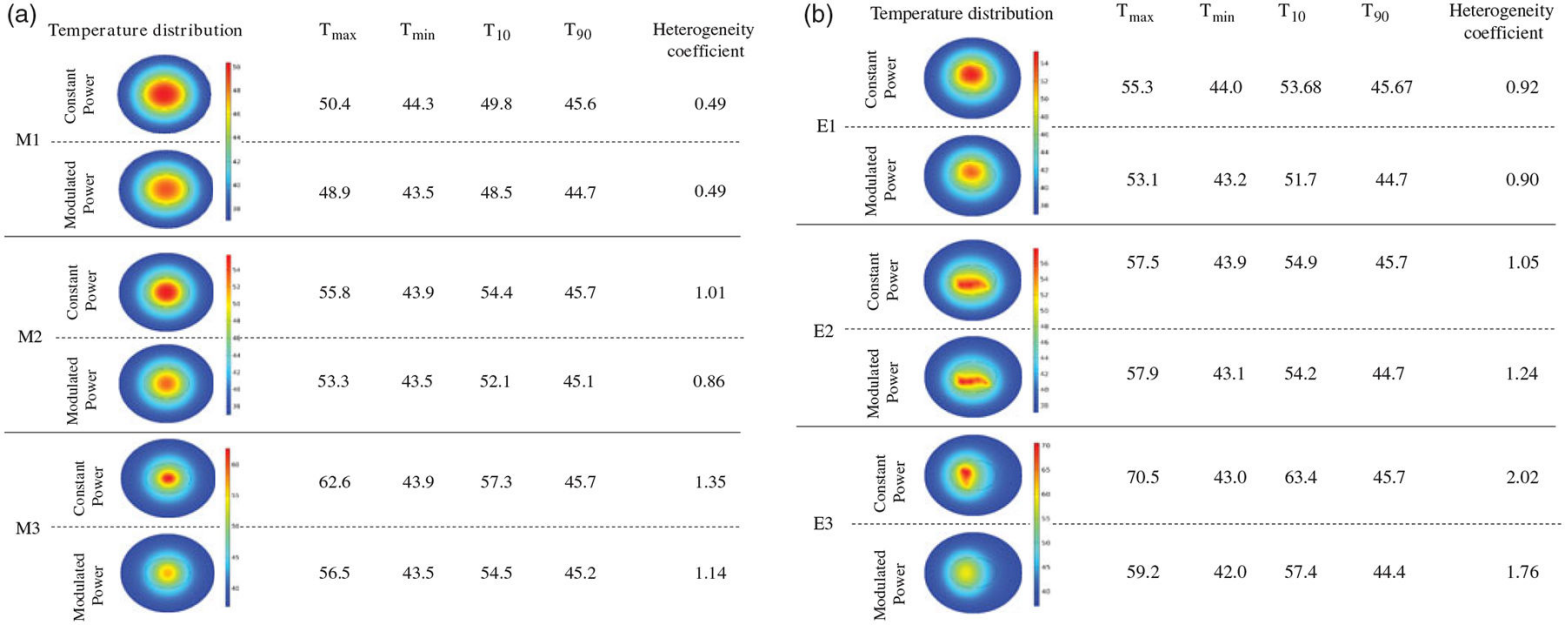
Modulated power heating reduces temperature heterogeneity when compared to constant power heating. Temperature distributions achieved in the tumor and healthy tissue after 20 min of heating by constant power and by power modulation with PID control using temperature feedback at the tumor-healthy tissue boundary for (a) ideal mathematical distribution models, (b) image derived nanoparticle distributions. *T*_max_ and *T*_min_ are the maximum and minimum temperatures inside the tumor. *T*_10_ and *T*_90_ are the temperatures achieved by at least 10% and 90% of tumor area and the dimensionless heterogeneity coefficient *HC* indicates the temperature heterogeneity relative to *T*_90_.

In Figures 5(a,b) we show temperature distributions and provide tabulated metrics comparing the two heating methods, while Figures S6 show temperature data obtained from each of the models after heating. For all simulated nanoparticle distributions, modulated power heating reduced both overall temperature and temperature heterogeneity. The latter was assessed by comparing *T*_10_ and *T*_90_, (temperature achieved with at least 10% and 90% of tumor area, respectively) and the dimensionless heterogeneity coefficient *HC* defined as *HC* = (*T*_10_ – *T*_90_)/(*T*_90_ – *T*_core_) [57]. For all models except E2, values of *T*_10_, *T*_90_, and *HC* decreased, signifying a more uniform temperature was achieved with power modulated heating.

**Figure 6.**
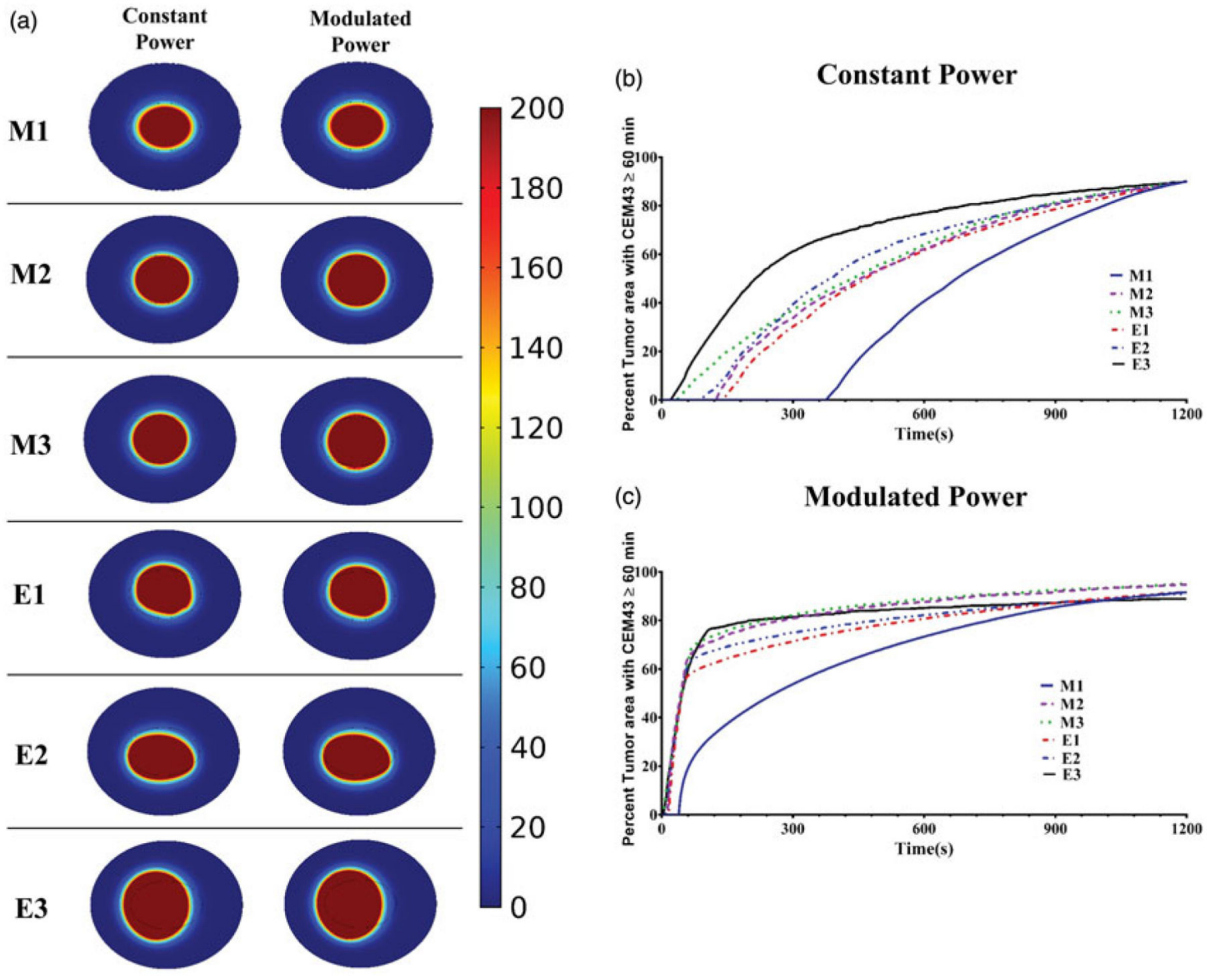
Power modulated heating using PID controller leads to faster therapeutic heating. (a) Thermal dose measured as CEM43 for the six distributions, during 20 min of simulated heating by constant isoeffect power (left column) and by power modulation with PID control (right column) using temperature feedback at tumor-tissue boundary with the modified Arrhenius perfusion model. (b) Time-dependent deposition of thermal dose, measured as CEM43 ≥ 60 min by constant (isoeffect) power heating; and, (c) with power modulated heating using PID control with temperature feedback at tumor-tissue boundary. The modified Arrhenius perfusion model was used.

The deposited thermal dose was comparable for both heating methods as illustrated in Figure 6(a). On the other hand, modulated power significantly decreased the time needed to heat >50% tumor area regardless of nanoparticle distribution, see Figures 6(b,c).

### PID controlled modulated power heating in 3D models

**Figure 7.**
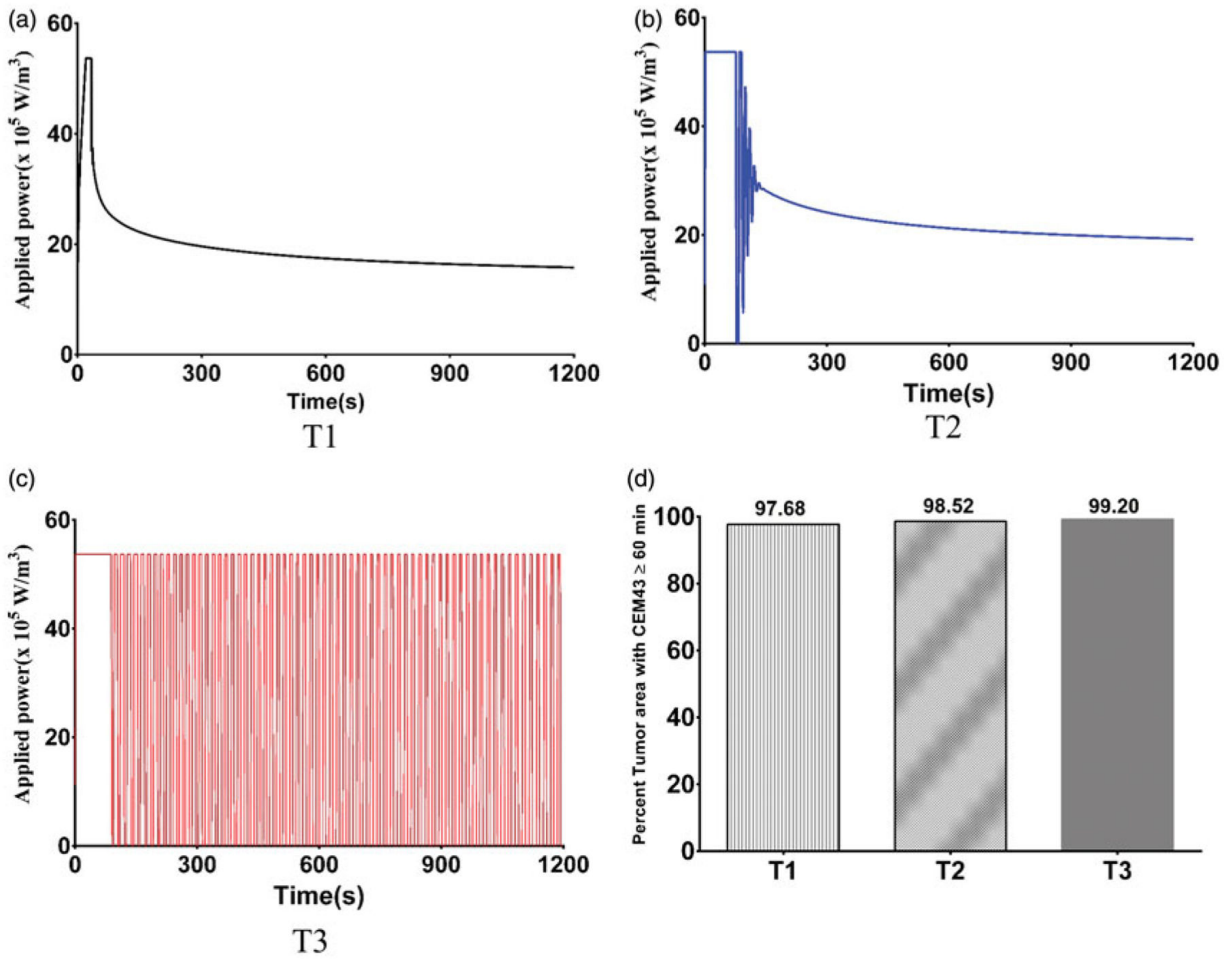
Variation of power with time for 3D models. Modulation of heating power with time using a PID control algorithm to achieve and maintain a target temperature of 43.5 °C at the probe location for (a) T1 – Uniform distribution, (b) T2 - Gaussian centered; (c) T3 – 3 pt Gaussian distribution mimicking 3-point nanoparticle injection; (d) percent of tumor area with CEM43 ≥ 60 min after 20 min of heating with PID controlled modulated power heating for the three 3D nanoparticle distribution models.

Extending the model to three dimensions (see Figures 3) provides additional insights into power-modulated heating, and better reflects scenarios likely to be encountered in either pre-clinical or clinical settings. In Figures 7(a-c), we display the time variation of applied power, and in Figure 7(d) the total tumor volume having achieved the target thermal dose. For the uniform nanoparticle distribution, T1, model, the applied heating power peaked and then reduced to a lower power (see Figure 7(a)), similar to its 2D counterpart. For the Gaussian-centered, T2, nanoparticle distribution model, PID control initiated application of maximum power followed by a brief period of damped oscillation stabilizing at a lower power (Figure 7(b)). Interestingly, the applied heating power oscillated continuously between maximum and minimum for the duration of heating for the 3-point Gaussian distribution, or T3, model (Figure 7(c)). All models exceeded the minimum 90% tumor volume CEM43 ≥ 60 min by the end of heating (Figure 7(d)).

**Figure 8.**
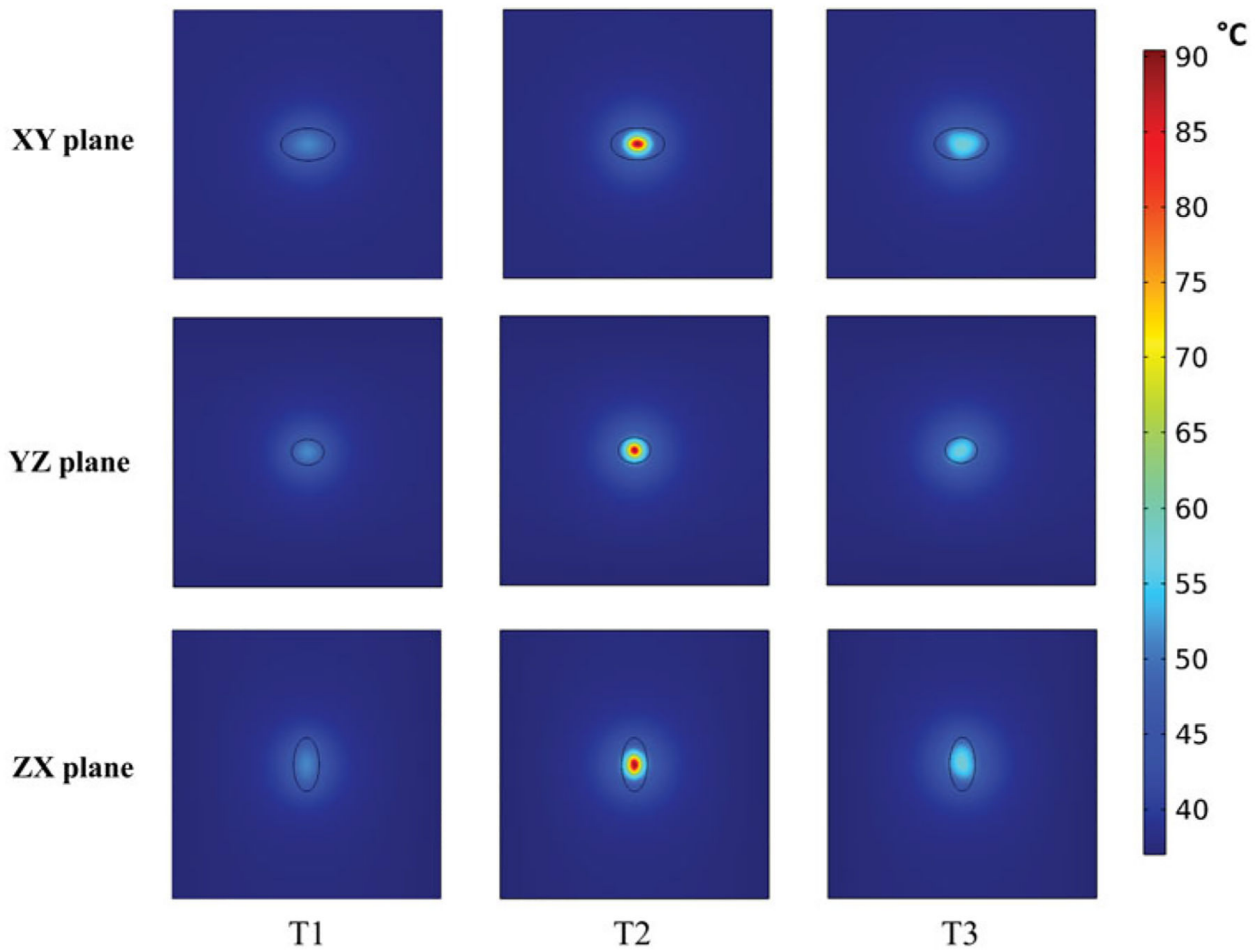
PID controlled power modulation achieves therapeutic temperatures within the tumor. Temperature distributions achieved inside the tumor and healthy tissue, (a) XY plane, (b) YZ plane, (c) ZX plane, for the three 3D nanoparticle distribution models T1, T2 and T3, after 20 min of heating by power modulation with PID control based on temperature feedback from probe at tumor-tissue boundary with the modified Arrhenius perfusion model.

Temperature distributions achieved in the tumor and healthy tissue along the XY, YZ, and ZX planes at the center of the tumor for the three 3D nanoparticle distribution models are shown in Figure 8. Regions with or adjacent to concentrations of nanoparticles achieved higher temperatures than did regions distant from the nanoparticles. The highest maximum intra-tumor temperature (91 °C) was predicted in the Gaussian-centered, or T2, model whereas the T1 (uniform) model achieved the lowest maximum temperature of 52 °C. Qualitatively, these results are similar to those observed with 2D distributions.

**Figure 9.**
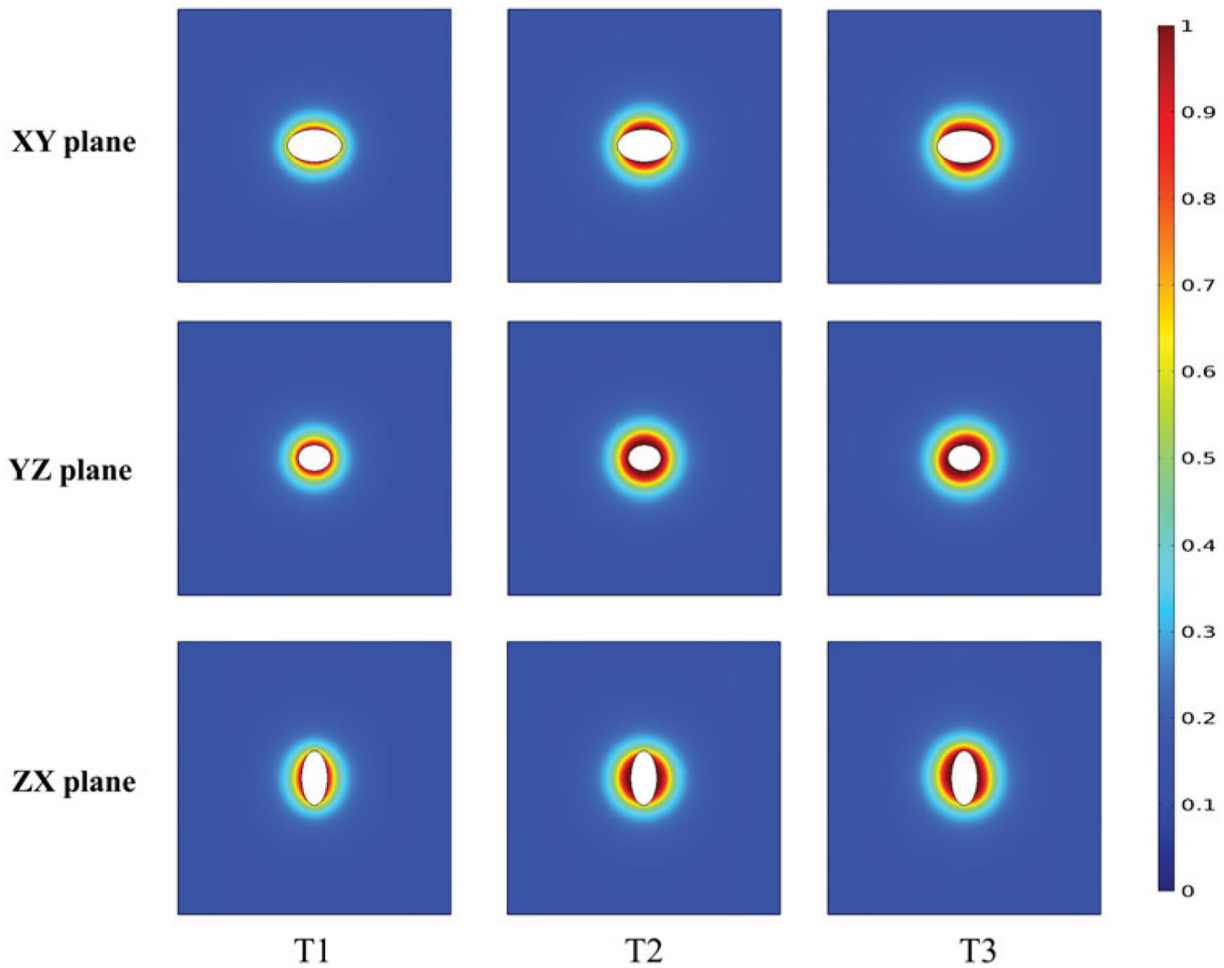
Healthy tissue damage is higher for regions closer to nanoparticle distributions. Degree of thermal damage (DS) achieved inside the healthy tissue, (a) XY plane, (b) YZ plane, (c) ZX plane, for the three 3D nanoparticle distribution models T1, T2 and T3, after 20 min of heating by power modulation with PID control based on temperature feedback from probe at tumor-tissue boundary with the modified Arrhenius perfusion model.

In Figure 9, we display predictions of damage to healthy tissue, given by the degree of stasis DS, for the 3D nanoparticle distribution models. It can be seen that the variation of damage in the healthy tissue depends on the proximity of the nanoparticles to the tumor-healthy tissue boundary.

## Discussion

The effectiveness of magnetic nanoparticle hyperthermia is largely determined by both nanoparticle distribution in tissues and heat transfer. Heat transfer in living tissues is a complex combination of heat conduction and capillary blood perfusion. Nanoparticle distributions depend upon tumor physical properties; and, significant variation is encountered even with convection-enhanced percutaneous delivery [14,58]. Thus, it is impossible to precisely control nanoparticle distributions for mNPH, but energy deposition can be controlled by adjusting the AMF. Computational modeling can be an efficient tool to explore various optimization strategies. In this study, we sought to explore various power modulation scenarios to identify promising strategies that can compensate for the variable and heterogeneous nanoparticle tissue distribution that accompanies mNPH.

We began with a comparison of six different nanoparticle distributions using 2D computational phantoms to enhance computational efficiency. Local concentrations of nanoparticles, positioned towards the tumor center and away from the tumor-tissue boundary, offer benefit to deposit significant energy to the tumor in a short time (see Figure 4). An idealized uniform nanoparticle distribution tends to generate the lowest local maximum tumor temperature because the nanoparticles are distributed throughout the tumor and heat generated by isolated nanoparticles rapidly transfers throughout the tissue yielding smaller gradients. Consequently, and perhaps not surprising – an idealized uniform nanoparticle distribution will require longer heating times to achieve a specific treatment goal. The benefit, however, is that risks to normal tissue are reduced with such a distribution because high-temperature gradients are less likely. Not surprising, constant power application with a constant perfusion model generated temperature profiles within tumors that closely approximated nanoparticle distributions, with ‘hot-spots’ manifesting where nanoparticle concentrations were highest (see Figure 4). With constant power, we compared the effects of constant perfusion with two temperature-dependent perfusion variations. Both of the variable-temperature perfusion models predicted higher tissue temperatures than did the constant perfusion model (see Figure S2). As perfusion, and associated heat removal decreased with increasing thermal damage more heat was retained in the tumor, leading to higher local temperatures and thermal dose.

A uniform temperature distribution in the tumor is often considered desirable; however, achieving a *minimum* effective temperature in 90% of tumor with sufficient control to preserve surrounding normal tissues is more realistic and is demonstrably effective [1,59]. Achieving a minimum clinically relevant thermal dose (CEM43 ≥ 60min) in a larger volume of tumor is considered a better measure of treatment outcome [1,10,51]. Equally important is minimizing the damage to surrounding normal tissues. The latter is often ignored, particularly in computational studies of mNPH. A reference isoeffect heating power, *Q*_iso_, was chosen as the minimum power required to realize a thermal dose in a maximum tumor area. The constant perfusion model underestimates tumor temperature(s), and consequently overestimates the power required to achieve a target thermal dose (Table 2) compared to temperature-dependent perfusion for different nanoparticle distributions

We combined non-linear temperature-dependent perfusion with power modulation of (non-linear) nanoparticle heating properties (see Figure S1) to reduce the impact of heterogeneous nanoparticle distributions in tissues. Attaluri et al. [14] previously showed with a 2D circular tumor model that modulating power using temperature feedback from a sensor at the tumor-tissue boundary can be used to achieve a minimal effective thermal dose (CEM43 T90). A non-spherical tumor shape and asymmetric nanoparticle distribution require more precise placement of temperature sensor. The criterion for choosing a probe location was to ensure that the thermal dose of CEM43 ≥ 60min was achieved in 90% of tumor area and in less than 5% of healthy tissue. It was possible to identify an ideal probe location for all nanoparticle distribution models, except E3 (Table 3). The concentration of nanoparticles close to the tumor-tissue boundary leads a predicted ineffective treatment and undesirable damage to healthy tissue.

We implemented a PID temperature controller (Figure 2(b)) to regulate power using temperature feedback from a probe located at the tumor-tissue boundary of our phantom models. The higher proportional (and consequently higher derivative) gains generated power oscillation or ‘cycling’ for all models having concentrated nanoparticle distributions (see Figure S3). For ‘uniform’ distributions M1 and E1, the applied power reached a maximum quickly then reduced to a lower power for the remainder of the treatment. For all other distributions, the applied power ramped quickly to maximum, and oscillated between maximum and minimum thereafter. For M3, E2, and E3, the peak of this oscillation was the maximum power possible *Q*_max_, while for M2 it was ~75% of *Q*_max_. The distance of the probe location from the nanoparticle heat sources for these distributions produced a delay between the control action and the temperature output. This required the controller to actively control the temperature output producing oscillatory input power. Arora et al. [53] reported similar oscillating control inputs in with their ultrasound treatments. Further tuning of the PID controller can improve its performance for more stable power application. Overall, our models predicted that PID controlled power modulation can improve tumor heating, regardless of nanoparticle distribution (see Figures S4 and S5), while simultaneously minimizing healthy tissue damage (Figure S4(b)).

A direct comparison of constant power and PID modulated power heating highlights the advantages of the latter. Overall, lower and more homogeneous temperatures were achieved for all models with power-modulated heating compared to constant power heating (Figure 5(a,b)). As expected, the uniform model (M1) showed no significant difference (~1%) between constant and power-modulated heating. The maximum benefit of modulated heating was observed for off-centered nanoparticle distribution (E3), where the maximum tumor temperature decreased by 16%. This can be potentially clinically significant, as previous studies have shown [10,60] that nanoparticle leakage and distribution occurs along the needle track, resulting in nanoparticle deposition close to tumor-healthy tissue boundary. Thus, even in cases where undesirable nanoparticle distributions occur, power modulation provides a better alternative over constant power heating to deliver effective thermal dose and in minimizing healthy tissue damage. With power-modulated heating, the time required to achieve therapeutic thermal dose in a large area of tumor (> 50%) is both considerably (~9x) shorter and less dependent on nanoparticle distribution than with constant power heating. Pulsed-power application may be the preferred method to perform mNPH because the time to reach therapeutic temperatures is short increasing the potential for effective therapy [61,62].

Following the optimization studies in 2D, we implemented the modulated power heating approach in 3D models. Results obtained demonstrate significant similarities to the 2D results. The PID controller generated a damped oscillating power during the first ~2min of heating for the Gaussian-centered nanoparticle concentration (i.e., T2, see Figure 3). This contrasts with trends observed in 2D results (M3, Figure S3). It is likely these differences occur because of different heat transfer between 2D and 3D models. An additional consideration, not assessed in the present work is the sensitivity of 3D models to variations of temperature probe placement. To reduce computational expense, 3D probe placement was chosen from our analysis of 2D scenarios which indicated the ideal placement of temperature probes was distal to nanoparticle localization. Our analysis of 2D models revealed greater sensitivity for inhomogeneous (i.e., concentrated, off-center) nanoparticle distributions. We expect that for 3D geometries such sensitivity may be heightened, warranting a further analysis for likely preclinical and clinical scenarios that have associated significant probe localization uncertainties. Nevertheless, we identify that a critical component to reduce such uncertainty is precise knowledge of nanoparticle localization.

Knowledge of the precise nanoparticle distribution, e.g., from imaging, would facilitate patient-specific optimized treatment planning by enabling computational modeling that incorporates active power modulation, and could be used to reduce uncertainties of temperature probe placement. Stated another way, the outcome of therapy can depend less on achieving a specific nanoparticle distribution within the tissue. A limitation of the current study was our use of a single temperature probe. Additional probes can potentially enhance power modulation to further optimize the simulated temperature distribution throughout the tumor; however, the invasive nature of probe-based thermometry may limit the number of probes used in clinical applications. Another limitation of our study was the exclusion of eddy current generation in tissues leading to off-target heating. A patient torso of ~30cm diameter exposed to AMF will display significant temperature rises from induced eddy currents, absent nanoparticles and depending upon the AMF conditions [63,64]. Additional study is warranted to explore further optimization by including these.

## Conclusions

To address one of the principal challenges of magnetic nanoparticle hyperthermia, we proposed modulated heating of magnetic nanoparticles using temperature feedback from the tumor-healthy tissue boundary, *in situ*, as a viable means to compensate for variable nanoparticle distributions in tissues. With both 2D and 3D models, we demonstrate it is possible to achieve effective tumor treatment with modulated power heating that realizes a more rapid rise to therapeutic temperature in a larger volume of tumor, a more homogeneous tumor temperature distribution, and with overall improved control of temperature in normal tissues. Further experimental investigation is warranted to develop this approach.

## Supplementary Material

Supplemental

## Figures and Tables

**Table 1.** Thermophysical properties used in simulations for the tumor, surrounding healthy tissue, and blood.

Properties	Tumour*	Tissuea	Blood^a,b^
Thermal Conductivity *(k*, W/m-K)	0.51 [41]	0.51 [41]	N/A
Density (*ρ*, kg/m^3^)	1045 [41]	1045 [41]	1060 [45]
Specific heat (*c*_p_, J/kg K)	3760 [41]	3760 [41]	3770 [45]
Initial Perfusion Rate (*ω*b_*i*_, 1/s)	0.0095 [42]	0.003 [42]	N/A
Metabolic Heat Rate (*Q*_m_, W/m^3^)	31872.5 [43]	6374.5 [43]	N/A
Pre-factor (*A*, 1/s)	1.8e^36^ [49]	1.03e^38^ [49]	N/A
Activation Energy (*E*_a_, J/mol)	2.38e^5^ [49]	2.49e^5^ [49]	N/A

a Numbers in square parentheses are citations to literature source, See References.

b Temperature of blood (*T*_*b*_) was fixed at 37 °C [51]. N/A, not applicable.

**Table 2.** Isoeffect heating power all nanoparticle distributions and three perfusion models.

	Isoeffective heating power (× 10^5^ W/m^3^)			Percent of healthy tissue with CEM43 ≥ 60min (%)		
Nanoparticle Distribution	Constant Perfusion	Arrhenius Perfusion	Modified Perfusion	Constant Perfusion	Arrhenius Perfusion	Modified Perfusion
M1	10.6	8.0	8.1	0.2	0.5	0.4
M2	11.8	8.0	8.1	2.0	2.0	1.9
M3	12.5	8.2	8.2	2.7	2.6	2.6
E1	13.6	8.8	8.9	6.6	5.1	5.2
E2	12.2	8.3	8.4	3.5	3.2	3.2
E3	19.6	10.5	11.0	18.4	13.8	14.5

**Table 3.** Percent area of tumor and healthy tissue having received a thermal dose CEM43 ≥60 min after 20 min of heating with two-step power modulation (Equation (10) based on temperature feedback from eight probe locations (P1–P8, see Figure 2).

	Tumour area with CEM43 ≥ 60min (%)					
Probe location	E1	E2	E3	M1^a^	M2^a^	M3
P1	89	94	96	91	94	94
P2	67	90	58	91	94	94
P3	86	31	59	53	56	54
P4	46	73	51	53	55	54
P5	93	68	88	72	79	79
P6	47	79	87	72	79	79
P7	77	55	38	72	79	79
P8	35	85	42	72	79	79
	Healthy tissue area with CEM43 ≥ 60min (%)					
P1	5	4	20	1	3	3
P2	1	3	1	1	3	4
P3	4	0	1	0	0	0
P4	0	1	0	0	0	0
P5	6	1	13	0	0	0
P6	0	2	13	0	0	0
P7	3	0	0	0	0	0
P8	0	3	0	0	0	0

a Symmetry was taken into account for probe locations.

**Table 4.** Open loop response and PID control parameters.

Model	*u*_ctrl_	Δ*T*(K)	*g*(K)	*τ*_1_(s)	*τ*_2_(s)	*f*(rad/s)	*ζ*	*K*_p_ (1/K)	*K*_*i*_ (1/(s.K))	*K*_d_ (s/K)
E1	0.3	6.4	21.4	3.6	213.7	2	1.0	10.2	0.047	33.47
E2	0.3	6.8	22.7	3.0	220.5	2	1.0	9.8	0.044	26.68
E3	0.3	5.3	17.6	10.4	253.1	2	1.0	14.9	0.057	145.54
M1	0.3	7.1	23.8	0.16	177.34	2	1.0	7.5	0.042	−0.67
M2	0.3	6.8	22.5	4.3	234.2	2	1.0	10.6	0.044	42.05
M3	0.3	6.7	22.4	6.6	227.9	2	1.0	10.5	0.045	64.63
T1	0.3	5.0	21.0	0.03	152.97	2	1.0	7.3	0.048	−1.63
T2	0.3	5.1	17.0	8.0	222.0	2	1.0	13.5	0.059	101.09
T3	0.3	4.7	15.8	7.3	232.7	2	1.0	15.2	0.063	103.72
