# Supplementary material for: Temperature-controlled power modulation compensates for heterogeneous nanoparticle distributions: a computational optimization analysis for magnetic hyperthermia
Source: Int J Hyperthermia. Author manuscript; Available in PMC 2019 Mar 11. (PMC6411438; doi:10.1080/02656736.2018.1538538)
Supplement: Supplemental [file NIHMS1005082-supplement-Supplemental.pdf]

Supplementary Materials

Temperature-controlled power modulation compensates for heterogeneous nanoparticle distributions: A computational optimization analysis for magnetic hyperthermia

Sri Kamal Kandala, Eleni Liapi, Louis L. Whitcomb, Anilchandra Attaluri, Robert Ivkov

Discretization of nanoparticle heating:

The heat rate per unit volume of tumour due to nanoparticles, Q_P (Equation 3 in manuscript text), if nanoparticles are present (=0 if nanoparticles are absent) is described by,

$$Q_P = Q_i \delta(\bar{x} - \bar{x_i}) \qquad \text{(S1)}$$

where Q_i is the heating power of the individual nanoparticle, and δ is the Dirac delta function, $\bar{x}$ denotes the spatial location (x, y) and $\bar{x_i}$ denotes the location of the nanoparticle (x_i, y_i).

$$\delta = \begin{cases} 1, if\ \bar{x} = \bar{x_i} \\ 0, if\ \bar{x} \neq \bar{x_i} \end{cases} \qquad \text{(S2)}$$

The total heating rate in the tumour due to nanoparticles, given by Q_{NP} (Equation 1 in the manuscript) can now be derived. Since the heating power of each nanoparticle is assumed to be same, Q_{NP} is given by ;

$$Q_{NP} = Q_i \sum_{i=1}^{N} \delta(\bar{x} - \bar{x_i}) \qquad \text{(S3)}$$

where N is the total number of nanoparticles. Integrating S3 across the entire tumour domain yields;

$$Q_{NP} = NQ_i \qquad \text{(S4)}$$

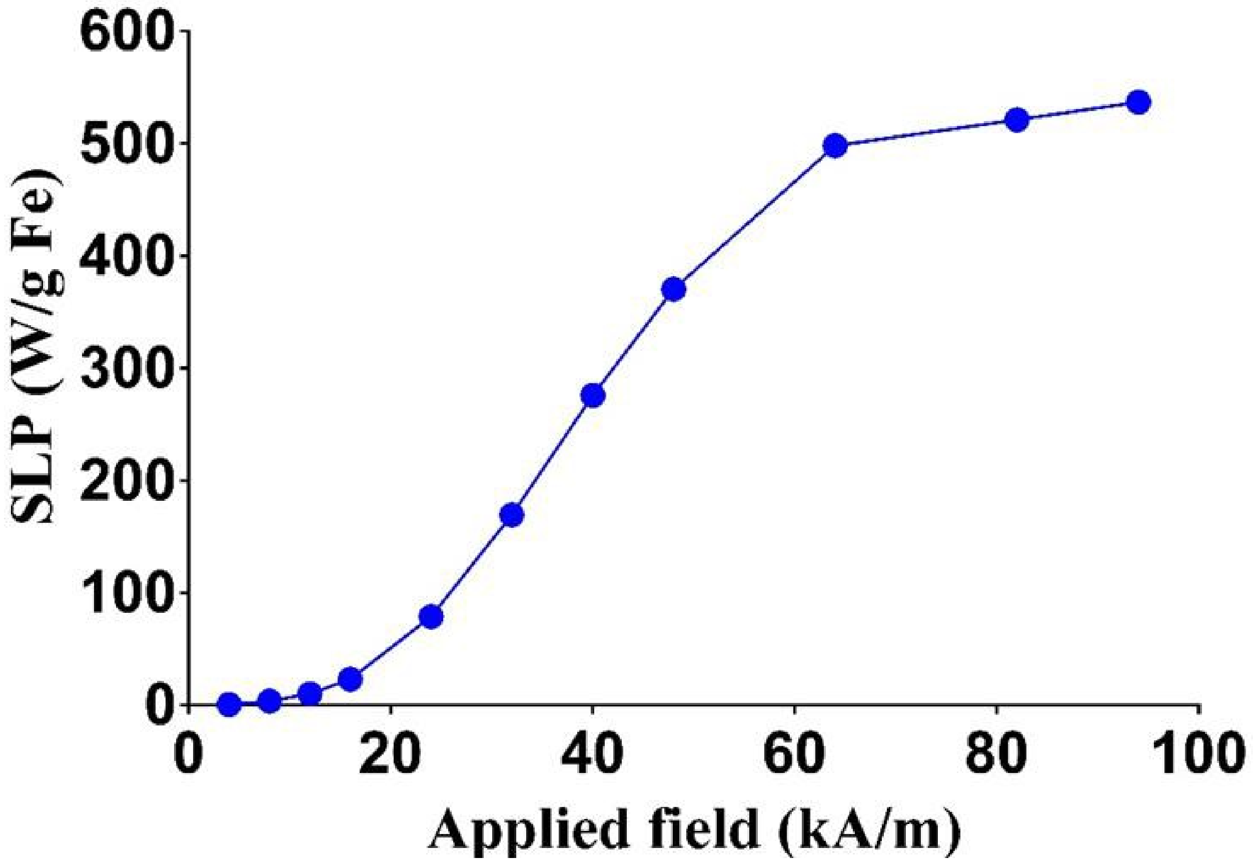

Figure S1: Specific loss power (W/ g Fe) versus applied field (peak, kA/m) for BNF-Starch nanoparticles [35] used as a representative nanoparticle for computational modelling. The heating performance, or specific loss power (SLP), corresponding to the value of the chosen individual heating power of the phantom nanoparticles in the text is 103.6 W/g Fe, which corresponds to peak magnetic field amplitude of ~13.5 kA/m. This assumes the total mass concentration of nanoparticles previously reported [14] was C_{Fe} = 5 mg Fe/cm^3 which was discretized from imaging data to yield N = 1460 ± 5 phantom nanoparticles using ~35 pixels/mm2 from imaging data (see text).

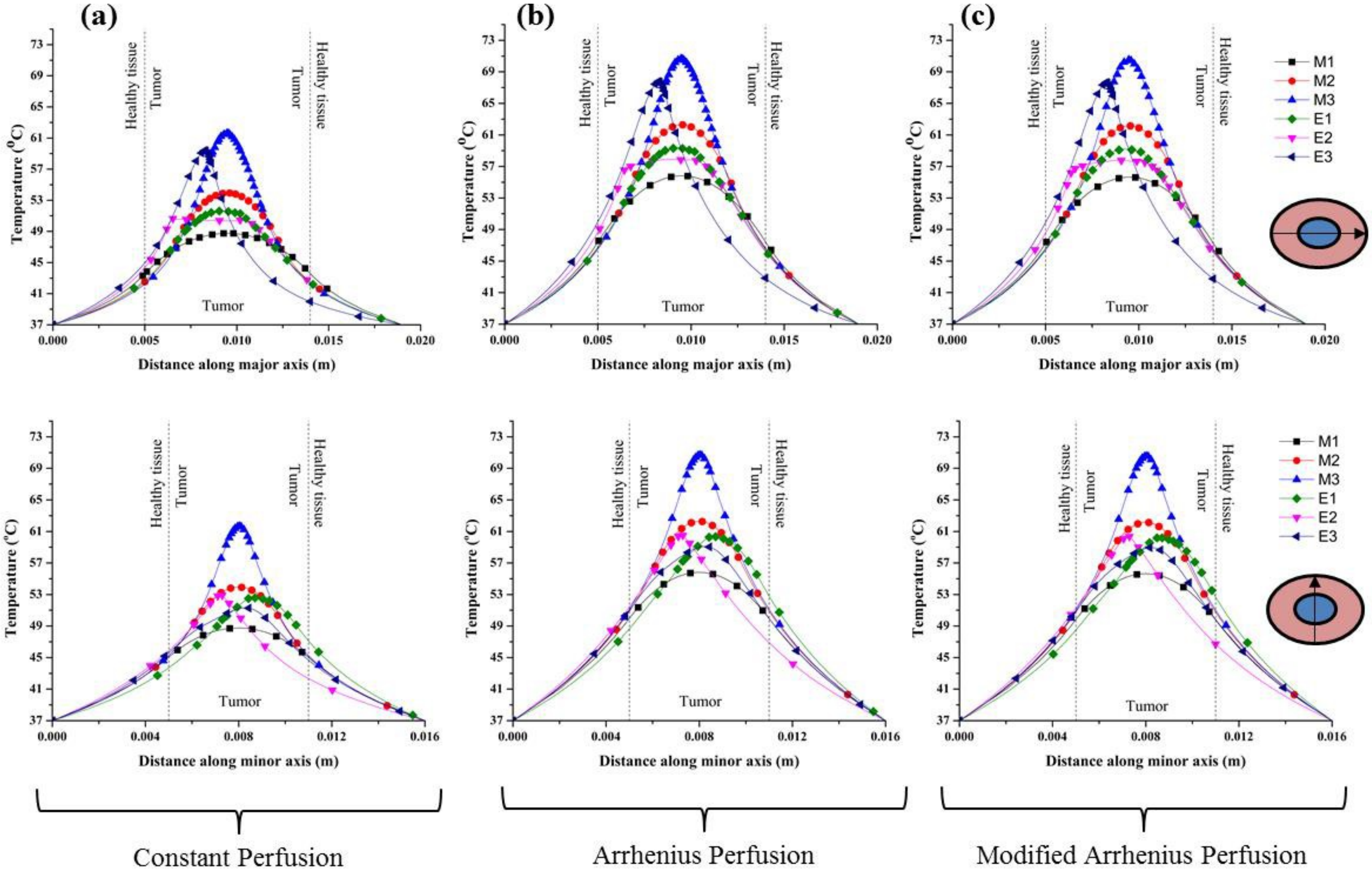

Figure S2: Choice of perfusion model influences model tumour and healthy tissue temperature distributions Temperature distribution along the major (upper panels) and minor (lower panels) axes of computational model, for the six nanoparticle distributions after 20 min of heating at constant power of Q_{NP} = 10.6 × 10^5 W/m^3, with (a) constant perfusion, (b) Arrhenius perfusion; and (c) modified Arrhenius perfusion. Model descriptions are provided in the text.

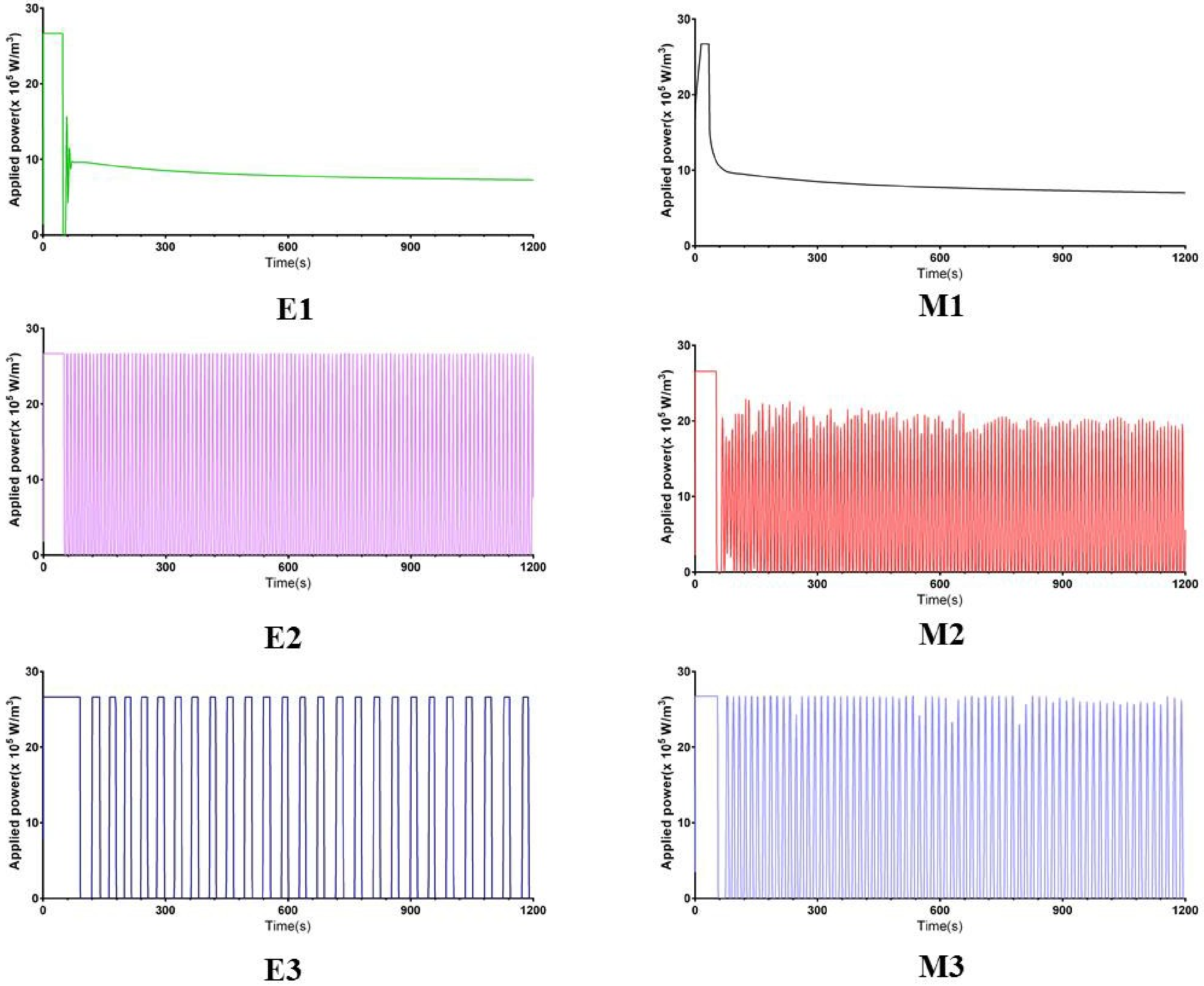

Figure S3: Compensating for variable nanoparticle distributions using power modulation demands variable power application. Heating power modulation using a PID control algorithm achieves and maintains a target temperature of 43.5°C at the probe location by adapting power modulation. Power application for (beginning on top left panel) E1- nanoparticles moderately uniform distribution; E2- concentrated distribution along the major axis; E3- concentrated and offset along the minor axis; M1- uniform; M2- uniformly concentrated in 40% of tumour area; and M3- Gaussian. Complete descriptions are provided in the text.

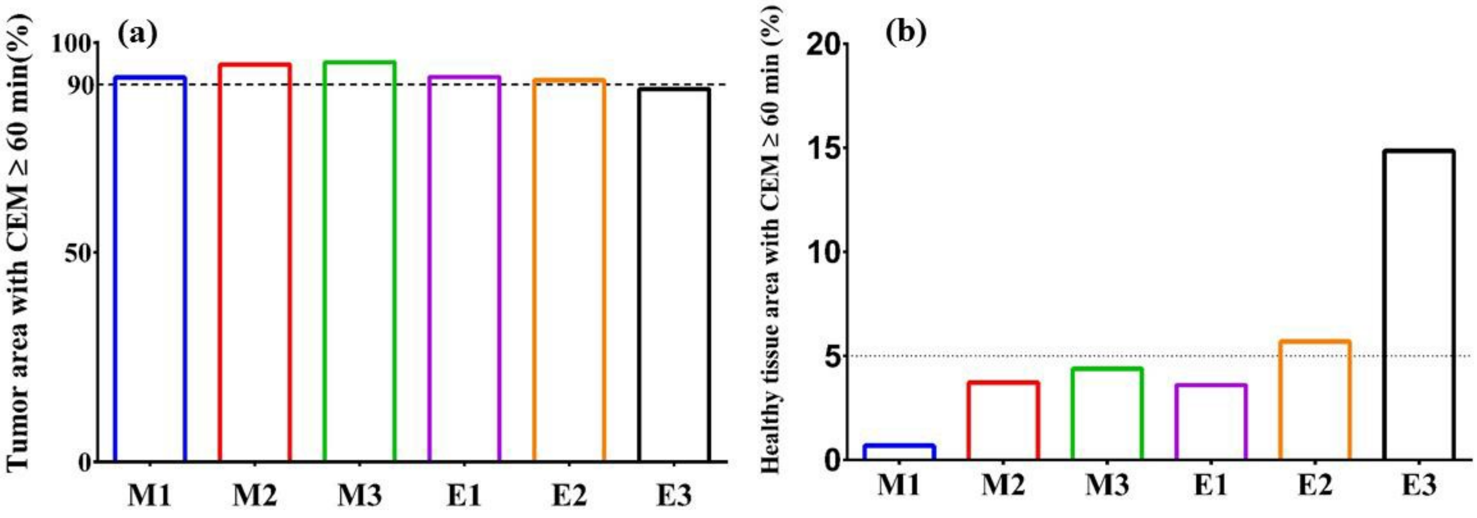

Figure S4: PID controlled power modulation achieves therapeutic thermal dose Percent of (a) tumour area, and (b) surrounding healthy tissue with CEM43 ≥ 60 min following 20 min of simulated heating with PID-controlled power modulation using temperature feedback for the modified Arrhenius perfusion model with all six nanoparticle distributions.

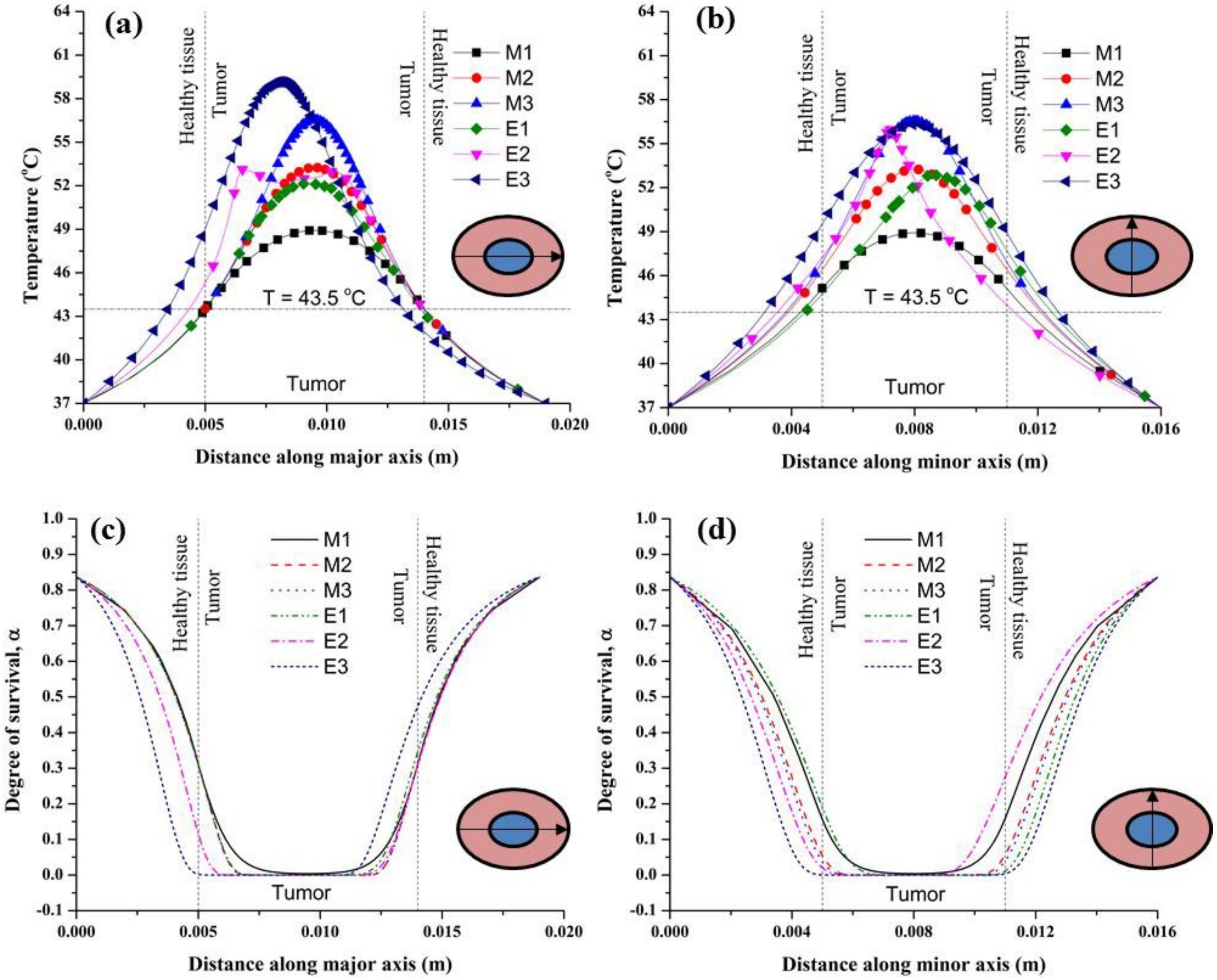

Figure S5: PID controlled power modulation achieves therapeutic temperatures within the tumour. Calculated values of (a) temperature distribution; (b) Degree of survival α, along the major and minor axes of computational model.

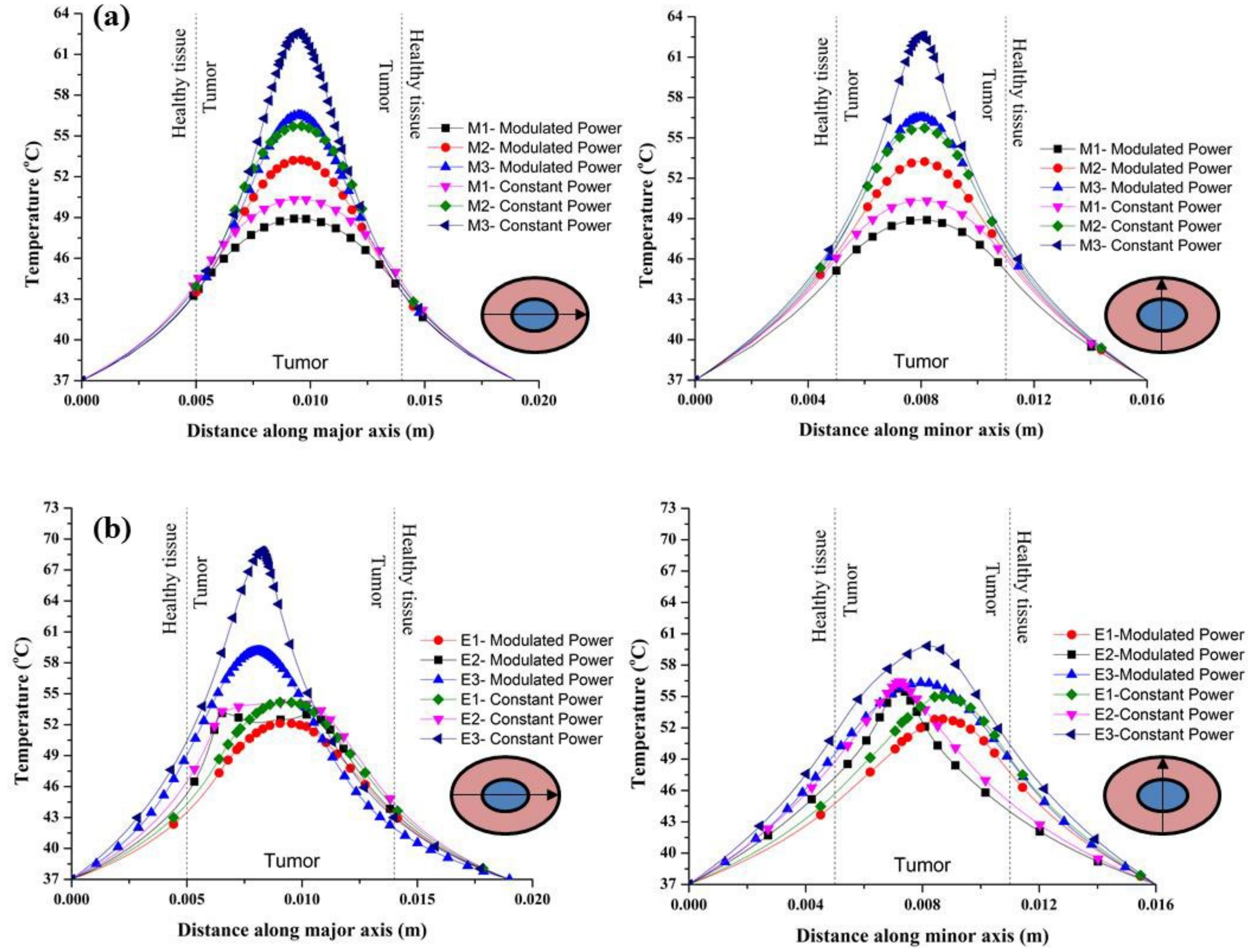

Figure S6: Power modulated heating using PID controller produced overall lower temperatures when compared to constant power heating. Temperature distributions along the major and minor axes for (a) ideal mathematical distribution models and, (b) image derived nanoparticle distributions, after 20 min of heating.

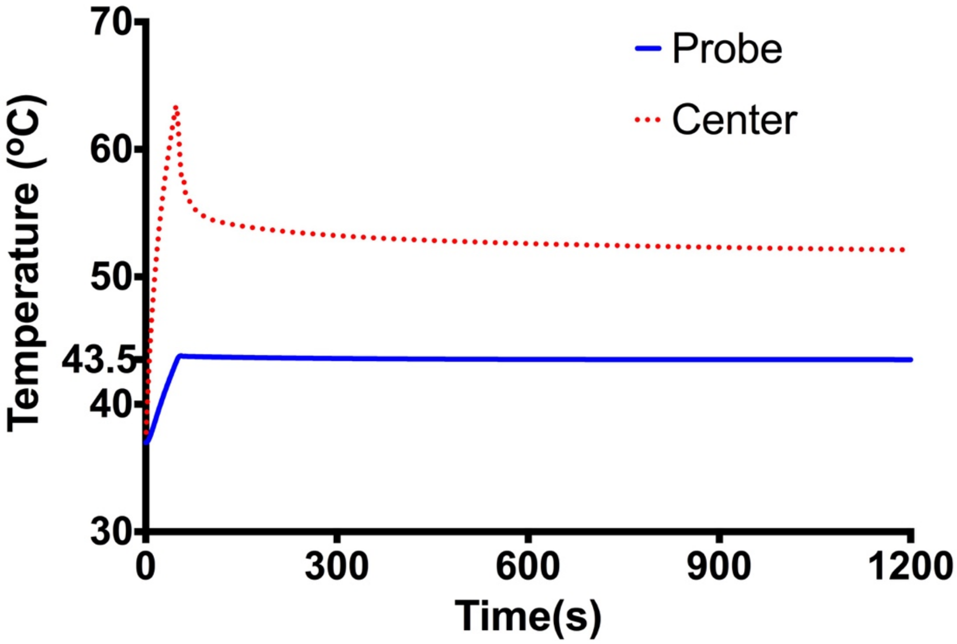

Figure S7: Variation of temperature with time at the center and probe location during power modulated heating using PID controller for E1 nanoparticle distribution.
